# Mercury anomalies and the timing of biotic recovery following the end-Triassic mass extinction

**DOI:** 10.1038/ncomms11147

**Published:** 2016-04-06

**Authors:** Alyson M. Thibodeau, Kathleen Ritterbush, Joyce A. Yager, A. Joshua West, Yadira Ibarra, David J. Bottjer, William M. Berelson, Bridget A. Bergquist, Frank A. Corsetti

**Affiliations:** 1 Department of Earth Sciences, University of Toronto, Toronto, Ontario M5S 3B1, Canada; 2 Department of Earth Sciences, Dickinson College, Carlisle, Pennsylvania 17013, USA; 3 Department of Geology and Geophysics, University of Utah, Salt Lake City, Utah 84103, USA; 4 Department of Earth Sciences, University of Southern California, Los Angeles, California 90089, USA; 5 Department of Earth System Science, Stanford University, Stanford, California 94305, USA

## Abstract

The end-Triassic mass extinction overlapped with the eruption of the Central Atlantic Magmatic Province (CAMP), and release of CO_2_ and other volcanic volatiles has been implicated in the extinction. However, the timing of marine biotic recovery versus CAMP eruptions remains uncertain. Here we use Hg concentrations and isotopes as indicators of CAMP volcanism in continental shelf sediments, the primary archive of faunal data. In Triassic–Jurassic strata, Muller Canyon, Nevada, Hg levels rise in the extinction interval, peak before the appearance of the first Jurassic ammonite, remain above background in association with a depauperate fauna, and fall to pre-extinction levels during significant pelagic and benthic faunal recovery. Hg isotopes display no significant mass independent fractionation within the extinction and depauperate intervals, consistent with a volcanic origin for the Hg. The Hg and palaeontological evidence from the same archive indicate that significant biotic recovery did not begin until CAMP eruptions ceased.

## Abstract (web summary)

The association between Central Atlantic Magmatic Province (CAMP) eruption volatiles and the end-Triassic mass extinction remains ambiguous. Here, the authors present mercury and palaeontological evidence from the same archive and show that significant biotic recovery did not begin until CAMP eruptions ceased.

## Graphical abstract

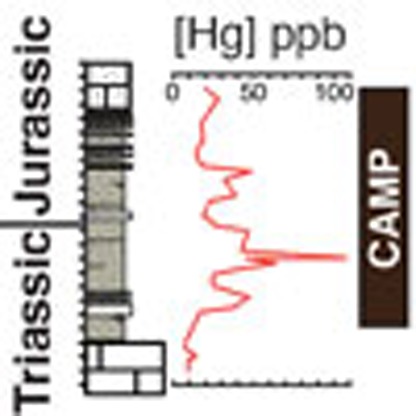

Various proxies reveal a dramatic rise in atmospheric *p*CO_2_ across the Triassic–Jurassic (T-J) boundary^1^,^2^ in association with the end-Triassic mass extinction ∼201.5 million years ago^3^,^4^ (for a summary of key biotic and geochemical events surrounding the T-J interval, see Supplementary Fig. 1). The extinction severely affected clades common to the modern ocean (the so-called Modern Fauna^5^) and reef-building scleractinian corals more significantly than any other extinction event and resulted in the lowest standing diversity in Phanerozoic time^6^. The robust coral reef ecosystem in the latest Triassic collapsed and reef/carbonate dwelling organisms were preferentially affected^7^, such that ocean acidification has been implicated in the extinction^8^,^9^,^10^. The extinction overlaps with the eruption of the Central Atlantic Magmatic Province (CAMP), a large igneous province emplaced as a result of the opening of the Atlantic during the rifting of Pangea^11^ (Fig. 1a) that was likely a significant source of CO_2_. High-resolution dating of CAMP basalts and sills from terrestrial successions in North America indicates that CAMP volcanism was geologically rapid and occurred in three or four pulses over ∼700 thousand years^3^ (Fig. 1b). Upper estimates of CO_2_ release to the atmosphere are ∼13 Gt CO_2_ per year (3 × 10^17^ mol CO_2_ released in discrete 1,000 year pulses)^2^, rivaling modern input rates (∼40 Gt CO_2_ per year). Although actual rates may have been somewhat lower, the T-J interval provides an opportunity to investigate the global consequences of a major carbon cycle perturbation.

Dated volcanic ashes that are interbedded with biostratigraphically important ammonites from deeper water strata in northern Peru^4^,^12^,^13^ demonstrate that the extinction was essentially coincident with the first major eruption of CAMP basalts at 201.51±0.15 Ma (Fig. 1b). The last pulse of CAMP basalts in North America occurred ca 200.9±0.064 Ma (ref. 3) and postdates the appearance of the earliest Jurassic ammonite *Psiloceras spelae* ca 201.39±0.14 Ma (ref. 4), hinting that biotic recovery may have been underway while CAMP was still erupting^3^. However, the palaeoenvironmental settings of the currently well-dated successions—terrestrial for CAMP, deeper marine for Peru—makes quantitative evaluation of the marine biotic recovery problematic, as most of the fauna typically used to assess ecological recovery occur in relatively shallow marine settings.

Mercury chemostratigraphy has recently been used to investigate the role of large igneous provinces in driving climatic events and biotic crises during mass extinctions^14^,^15^,^16^,^17^,^18^,^19^. Large volcanic events may cause substantial short-term increases to the atmospheric Hg burden, and the long residence time of Hg^0^_(g)_ in the atmosphere (∼1 year) makes it possible for large volcanic Hg fluxes to have global impacts^20^. Once released to the atmosphere, Hg may undergo complex cycling (for example, transformation, deposition, re-emission) before long-term burial, which occurs dominantly in marine sediments on million-year timescales^21^. Mercury primarily enters marine systems through direct atmospheric deposition or through riverine inputs, which transfer terrestrial Hg (derived from crustal and also atmospheric sources) to coastal sediments^21^. Terrestrial Hg of atmospheric origin may potentially be transported to coastal sediments bound to organics or clay particles^22^. In the ocean, organic carbon is a major scavenging pathway and sink for Hg, and marine sediments often preserve the strong association between Hg and organic matter^23^.

An exemplary T-J shallow shelf succession deposited between the Sierran arc and the North American continent in eastern Panthalassa allows detailed analysis of local palaeoecological recovery that can be tightly correlated to worldwide biotic trends. The section is well-exposed in Muller Canyon of the Gabbs Valley Range, Nevada^24^ (Fig. 1), but has lacked detailed correlation with CAMP volcanism. Here, we investigate Hg concentrations and Hg isotopes in continental shelf sediments from Muller Canyon as tracers of volcanism and combine Hg measurements with palaeoecological data from this succession to assess the timing of marine biotic recovery relative to the eruption of the large igneous province. We also measure the amount of total organic carbon (TOC) to determine if variation in Hg concentrations are driven by lithological changes controlling organic matter content and provide organic carbon isotopic measurements in order to directly tie trends in Hg to globally recognized carbon isotope excursions across the T-J interval. In Muller Canyon, Nevada, mercury anomalies (as indicated by both Hg and Hg/TOC levels) appear in the extinction interval and persist in association with a depauperate (low diversity) early Jurassic fauna. They are not observed in pre-extinction strata, and Hg levels fall before significant pelagic and benthic faunal recovery begins. Furthermore, no significant mass independent fractionation (MIF) of Hg isotopes is present within the extinction and depauperate intervals, consistent with a volcanic origin for the Hg anomalies, versus small MIF in adjacent strata. Based on Hg concentrations and Hg isotope chemostratigraphy, we conclude that eruptions from CAMP ceased before significant biotic recovery was underway.

## Results

### Organic carbon isotopes

Our δ^13^C_org_ measurements (Fig. 2 and Table 1) are consistent with previous studies^25^,^26^,^27^,^28^ (Supplementary Fig. 2) and reveal a negative δ^13^C_org_ excursion coincident with the extinction interval (termed the initial carbon isotope excursion or I-CIE (ref. 29)), followed by a positive excursion. We display our carbon isotope data in Fig. 2 for direct comparison to our new Hg chemostratigraphy and provide the fault-corrected data from Ward *et al*.^27^ in Supplementary Fig. 2 for comparison. Although minor differences exist between the various δ^13^C_org_ profiles (see Guex *et al*.^26^), all δ^13^C_org_ profiles broadly record a negative δ^13^C_org_ excursion in association with the extinction interval, followed by a positive excursion in association with *P. spelae*.

### Palaeoecology

The uppermost Triassic strata of the Mount Hyatt Member of the Gabbs Formation represent a prolific, bivalve-dominated Late Triassic carbonate ramp assemblage^30^ (Figs 2 and 3). The shift to siliciclastic-dominated sedimentation in the overlying Muller Canyon Member signifies a collapse of the vibrant carbonate system in association with the mass extinction^24^, which is marked by the last occurrence of the Triassic ammonite *Choristoceras crickmayi* and is coincident with the onset of the negative δ^13^C_org_ excursion^25^,^26^,^27^,^28^. The first occurrence of *Psiloceras spelae* (official marker of the basal Jurassic) above the last occurrence of *C. crickmayi* brackets a 7-m-thick extinction interval within the Muller Canyon Member, which records benthic fossils limited to rare microscopic gastropods and sponge spicules^24^. Depauperate benthic macrofauna in the upper 10 m of the Muller Canyon Member comprise minor bioturbation^31^ and rare bivalves (for example, *Agerchlamys*^27^ and *Modiolus*^32^, also found in the immediate extinction aftermath in England^33^ and Austria^34^), which occur in tandem with a minor increase in ammonoid diversity^25^ (cosmopolitan genera ubiquitous across Panthalassa^12^,^25^,^35^; Figs 2 and 3). The first phase of ecological recovery following the depauperate interval is indicated by a substantial increase in ammonoid diversity^12^,^25^ in the pelagic realm and the appearance of a pervasive demosponge-dominated ecosystem in the benthic realm^36^,^37^ (recovery state 1 in Fig. 3). The early recovery phase represents an ecological state shift (*sensu* Hull *et al*.^38^), which is also recorded in Peru^37^ and Austria^39^. Trophic complexity matching or surpassing pre-extinction conditions^40^ and a return to carbonate-dominated benthic biota^36^, including the first North American Jurassic corals^41^, did not occur until ∼2 million years after the extinction (recovery state 2 in Fig. 3).

### Mercury and organic carbon concentrations

Sediments of the Gabbs and Sunrise Formations have low organic carbon contents (<0.5% TOC) that do not correlate with Hg concentrations (Fig. 2 and Supplementary Fig. 3). First-order structure in Hg and Hg/TOC reveals a rapid rise and peak within the extinction interval, with a few smaller peaks in the overlying depauperate interval (Figs 2 and 3). Significant biotic recovery occurs as Hg concentrations return to pre-extinction levels^36^,^37^ (see recovery state 1 in Fig. 3). The decoupling of Hg and organic carbon within these strata suggests that variations in Hg content primarily result from changes in Hg loading to coastal waters rather than changes in the size of the organic carbon sink. It also implies that sources of Hg and organic carbon to these strata were, at least in part, different. Strata with the highest Hg/TOC in the Muller Canyon section have ∼600 p.p.b. of Hg per %C_org_, far more Hg per unit C_org_ than found in many contaminated sediments today (for example, average p.p.b. of Hg per %TOC in contaminated sediments measured in San Francisco Bay is ∼200 (ref. 42)).

### Mercury isotopes

We use Hg isotopic analyses to further explore the sources of Hg in the Muller Canyon succession. Mercury isotopes undergo large mass-dependent and mass-independent fractionations in nature and can be used to trace Hg sources and cycling (see Blum *et al*.^43^ and references therein). We report mass dependent fractionation (MDF) using δ^202^Hg values (see Methods for Hg isotope nomenclature). δ^202^Hg values of the Muller Canyon strata are primarily negative and range from −1.78 to 0.12‰ (see Table 1, Supplementary Fig 2 and Supplementary Table 1 for details). Because MDF can result from many physical, chemical and biological reactions, we do not interpret MDF signatures here. However, negative δ^202^Hg values are typical of both marine sediments and volcanic emissions. To investigate whether or not the Muller Canyon Hg anomalies were derived from CAMP volcanism, we focus on Hg-MIF signatures of these strata. Because Hg-MIF is primarily associated with photochemistry in natural samples and occurs during far fewer pathways than MDF (see Blum *et al*.^43^ for a summary of MIF and MDF pathways), it is unlikely that Hg-MIF signatures are altered by post-depositional processes.

Modern Earth surface environments, including marine sediments, often carry measurable amounts of odd isotope Hg-MIF (for example, see Yin *et al*.^44^), which is thought to result from the aqueous photochemical cycling of Hg (ref. 45). In comparison, direct isotopic measurements of volcanic Hg emissions display no measureable MIF^46^, and measurements of igneous rocks, ores and most hydrothermal precipitates also provide no evidence for Hg-MIF in solid Earth materials, except in contexts where sedimentary/surface Hg may be leached or recycled by geologic processes^43^.

Odd-isotope MIF (as indicated by Δ^199^Hg values in Fig. 2; see the Methods for Hg isotope nomenclature) is present in two of the lowermost samples analysed (both part of prolific Triassic carbonate ramp) and re-appears at the top of the depauperate interval where Hg is at background levels (Fig. 2 and Supplementary Fig. 2). Out of the seven samples analysed for isotopes below and above the extinction/depauperate intervals, five display measureable MIF, with Δ^199^Hg values ranging from 0.11 to −0.30‰ (Fig. 2, Table 1 and Supplementary Fig. 2). The odd-isotope MIF signatures present in the lower and upper-most intervals are consistent with those observed in modern coastal and oceanic sediments (for example, see Yin *et al*.^44^ and references therein) and suggest some similarity between modern and ancient Hg cycling. In contrast, no significant Hg-MIF is recorded throughout the extinction and depauperate intervals, when increases in Hg and Hg/TOC are observed (Fig. 2). The total range of measured Δ^199^Hg values within these intervals is −0.05 to 0.07‰ (Table 1), and thus most values fall within experimental error of zero. We interpret the paucity of MIF as evidence for a significant influx of volcanic Hg from CAMP during these periods.

## Discussion

**Figure 1:**
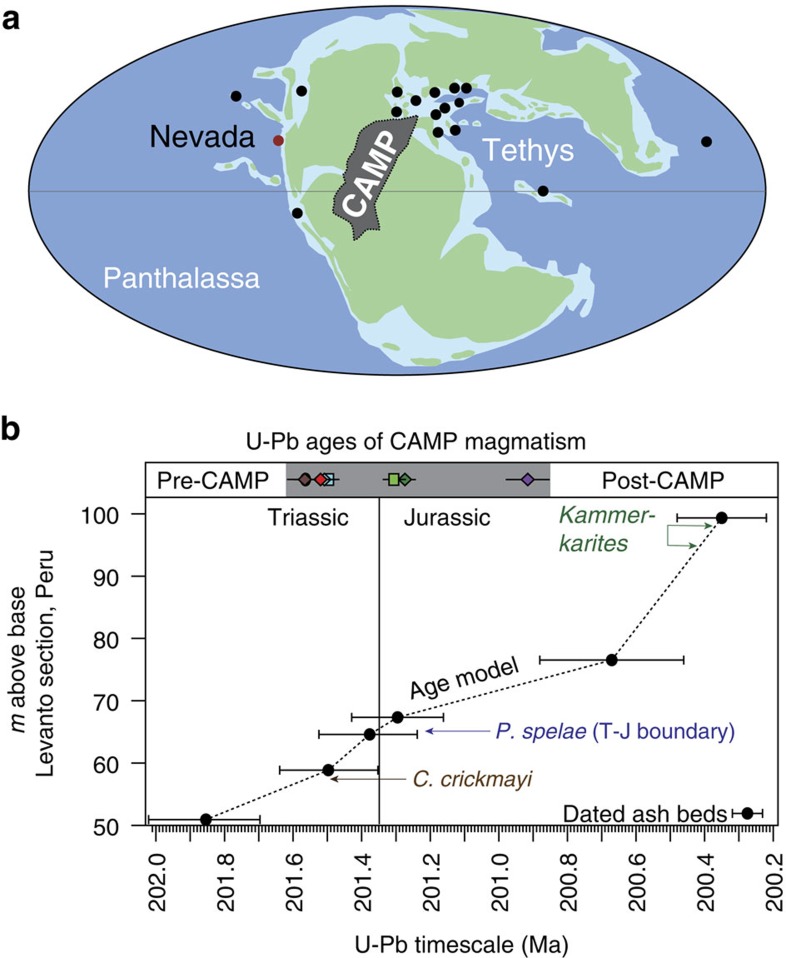
Geography of the T-J interval and age model. (**a**) Palaeogeographic map of the T-J interval showing the hypothesized extent of CAMP with the study site and other key T-J localities marked (with a red dot and black dots, respectively), modified from Greene *et al*.^8^ (**b**) Comparison of ammonoid biostratigraphy and U-Pb dates from interbedded volcanic ashes in Peru^4^,^13^,^25^ and U-Pb ages for CAMP magmatism largely from N. America and Morocco^3^ (top grey bar). The initiation of CAMP volcanism is coincident with the end-Triassic extinction (typically indicated by the last occurrence of *C. crickmayi*) and continues through the earliest Jurassic.

**Figure 2:**
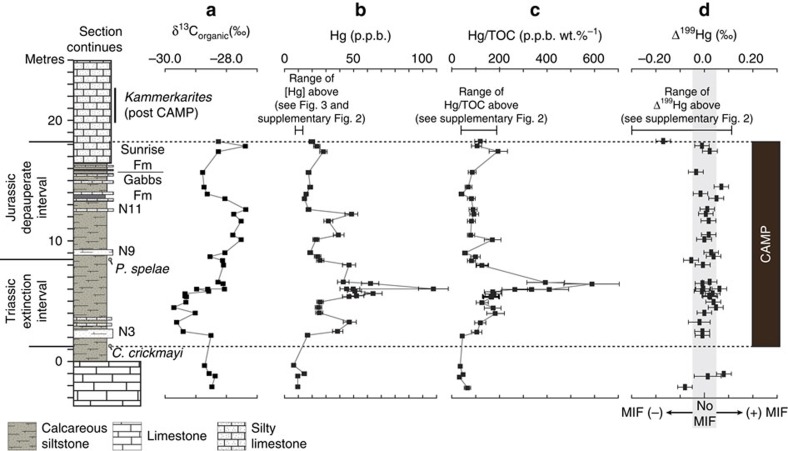
Mercury and organic carbon geochemistry of T-J interval, Muller Canyon, Nevada. Plots show (**a**) δ^13^C_org_, (**b**) Hg, (**c**) Hg/TOC and (**d**) Δ^199^Hg for Muller Canyon, Nevada, along with lithology and key ammonites. Marker beds N3, N9 and N11 are after Guex *et al*.^25^ In panel **b**, error bars on Hg are 2 s.d. In panel **c**, error bars represent the combined error on Hg (2 s.d.) and TOC (1 s.d.) concentration measurements. In panel **d**, errors on individual Δ^199^Hg measurements are as reported in Supplementary Table 1 and represent either 2 s.e.m. of sample replicates or 2 s.d. of the JT Baker standard, whichever is higher (see the Methods for further explanation). The vertical grey bar in panel **d** is centred on Δ^199^Hg=0.00‰ and extends from −0.05‰ to +0.05‰. Samples for which the Δ^199^Hg values and associated error bars fall within the shaded grey region can be considered to have Δ^199^Hg values within experimental error of zero, and thus no measureable mass independent fractionation (MIF).

**Figure 3:**
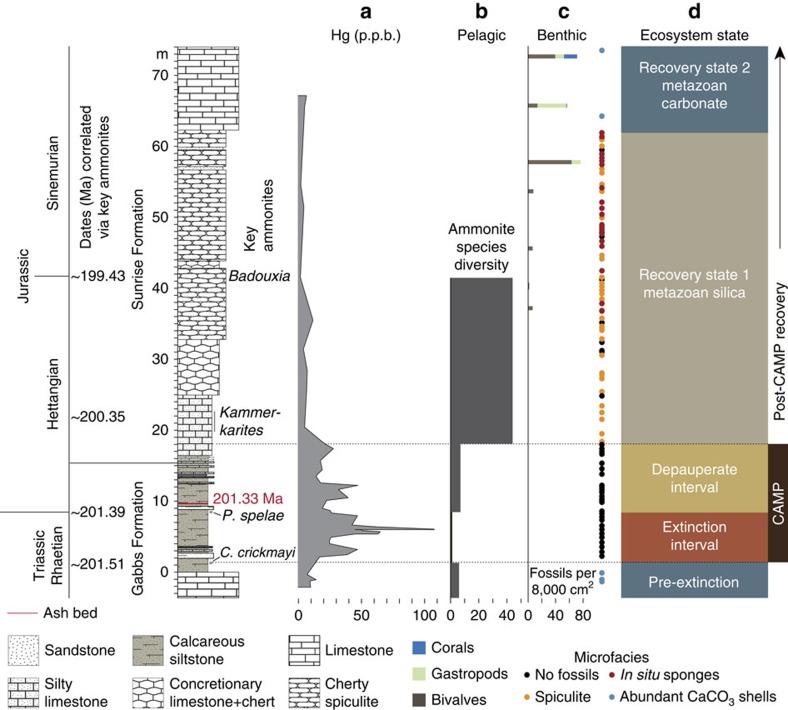
Summary of key features of the T-J interval, Muller Canyon, Nevada. Panels compare (**a**) Hg chemostratigraphy (this study), (**b**) ammonite species diversity from Guex *et al*.^25^; (**c**) benthic palaeoecology and microfacies modified from Ritterbush *et al*.^36^; and (**d**) ecosystem state for Muller Canyon, Nevada, in association with lithology and key dates. Nevada ash date is from Schoene *et al*.^13^ and approximate ages of key ammonites are from Wotzlaw *et al*.,^4^ and Schoene *et al*.^13^ (see Fig. 1b). These comparisons show that significant biotic recovery follows the Hg anomalies and provide evidence that biotic recovery began after the cessation of CAMP magmatism.

If the mercury anomalies within the extinction and depauperate intervals are from volcanism as isotopes suggest, then significant biotic recovery did not begin until eruptions associated with CAMP ceased. The preservation of a volcanic Hg isotopic fingerprint in sediments distant in location from the CAMP eruptions (Fig. 1) implies that Hg emissions from CAMP dominated the Hg pools at Earth's surface and altered the Hg cycle such that an insufficient amount of Hg released by volcanism underwent the aqueous photochemical transformations necessary to impart significant MIF. We have also considered the possibility that the input of volcanic Hg could be derived from arc volcanism proximal to the Muller Canyon succession, as indicated by the presence of an ash in the section. However, Hg concentrations in the strata closest to the ash layer (9.5 m) are relatively low (18.5–22.5 p.p.b.) when compared with other strata within the extinction and depauperate intervals (Fig. 2). It thus seems unlikely that Hg signals from arc volcanism are resolvable within the resolution of our sampling or could explain the elevated Hg levels on the timescale represented by the Muller Canyon succession. This interpretation is also supported by evidence that aquatic sediments do not reliably archive short-term Hg releases associated with sporadic large explosive eruptions^21^.

In summary, we show for the first time that Hg concentrations and isotopic compositions record the timing of massive volcanism in a marine section that spans the T-J interval, strengthening the case for CAMP's potential role in the mass extinction. Robust biotic recovery, which initially occurred in the form of bio-siliceous deposition^36^, did not begin until Hg concentrations returned to pre-extinction levels and Hg-MIF re-appeared, indicating the cessation of major CAMP volcanism. This inferred timing of recovery contrasts with previous suggestions^3^ that the recovery was underway as CAMP was still erupting.

Ocean acidification via CO_2_ input from CAMP has been suggested as a potential kill mechanism for the end-Triassic extinction^9^. An initial lowering of carbonate saturation may have contributed to the extinction of carbonate biota, but the Nevada section reveals that the carbonate-dominated ecosystems did not recover for nearly 2 million years after the extinction and ∼1 million years after the cessation of CAMP volcanism. Ocean acidification models typically predict much shorter recovery time scales (typically 10–100 ka)^10^, suggesting that ocean acidification alone cannot explain the prolonged disruption of metazoan carbonate-dominated ecosystems in the aftermath of the end-Triassic mass extinction. Other factors (for example, the initial shift in ecological state dominated by siliceous sponges, among others) may have played a role in the pattern of carbonate recovery^37^. Whatever the case, our new data from Nevada suggest that the long process of biotic recovery began in earnest once CAMP volcanism drew to a close.

## Methods

### Carbon measurements

Samples were collected from the field following the stratigraphy of Guex *et al*.^25^,^26^ (see Supplementary Fig. 4 for an image of the collection site). Samples were inspected and those with veins and weathered surfaces were removed. Samples were crushed in a jaw crusher and then pulverized in an agate ball mill at the University of Southern California. An aliquot of powder from each sample (∼0.5 g) was dissolved in 40 ml of 1 M hydrochloric acid and heated to 70 °C for 4 h to remove all carbonate mineral phases. This method is similar to that described by Ward *et al*.^27^. Samples were washed with deionized water three times and dried at 50 °C.

Weight percent organic carbon was determined on decarbonated powder using a Picarro cavity ring down spectrometer (G2131-i) coupled via a Picarro Liason (A0301) to a Costech Elemental Combustion System (EA 4010). This determination of organic carbon content was converted to a value of % TOC taking into account the amount of carbonate loss during acid treatment. Errors were calculated by replicate analyses of samples and standards. The 1 s.d. uncertainty was assigned as 10% of the reported value, which takes into account uncertainties associated with decarbonation. Standards included both internal CaCO_3_ standards and the USGS-40 reference material (L-glutamic acid).

The isotopic composition of organic carbon was also determined using the Picarro cavity ring down spectrometer and is reported in delta notation (δ^13^C_org_) relative to the Vienna Pee Dee Belemnite standard. The uncertainty on the δ^13^C_org_ values was assessed from replicate runs of standards (including NBS-18 calcite, USGS-40 and internal carbonate standards) and samples. Replicate analyses were run on 33% of the samples. Standard deviation on replicate analyses was on average <0.1‰. Uncertainties and blanks associated with this methodology are further discussed in Subhas *et al*.^47^

### Mercury concentration measurements

Samples were inspected, crushed and pulverized at the University of Southern California, as described above. Total Hg was measured using a Hydra II_c_ Direct Mercury Analyzer (Teledyne Leeman Labs) at the University of Toronto. Within the Hydra II_c_, samples were combusted in two stages under an oxygen flow of 350 ml min^−1^. First they were heated to 300 °C for 30–60 s, and then decomposed at 800 °C for 300–500 s. After combustion, the evolved gases were carried through a heated catalyst tube to remove possible interferences (for example, halogen compounds, sulfur oxides, nitrous oxides) and Hg was captured on a gold amalgamation trap while combustion gases were removed from the detection cell. The gold trap was then heated for 30 s at 600 °C to release Hg. Hg was carried to the detection cell where absorbance from a mercury lamp was measured at 253.7 nm.

Calibration was performed using a fresh, gravimetrically prepared NIST 3133 Hg standard in a 0.25% L-cysteine solution. Blank absorbance was <2% of typical sample signals and always <4%. Sample boats were periodically re-combusted to check that all available Hg had been released during the initial analysis. To determine measurement precision, the NIST 3133 L-cysteine solution was periodically combusted and analysed alongside samples. The measured concentrations of the NIST 3133 standard are within 5% of nominal values.

Samples measured more than once are reported as the mean of duplicate measurements (Supplementary Table 1). Reproducibility of sample concentrations was better than 10%. To check measurement accuracy, powders of NIST SRM 1944 (New York/New Jersey Waterway Sediment) and NIST SRM 1646a (Estuarine Sediment) were repeatedly combusted over the period of sample analysis. The average value for NIST 1944 was 3,496±334 p.p.b. (2 s.d., *n*=2), which is within the certified value of 3,400±500 p.p.b., and the average value for NIST 1646a was 27.7±2.8 p.p.b. (2 s.d., *n*=9). Although NIST 1646a is not certified for Hg, we used it as in-house external standard because our batch had a similar Hg content to the samples. The measured concentrations of NIST 1646a are consistent with the long-term values obtained on this standard in our laboratory. Based on the reproducibility of samples and external standards, errors on Hg concentration measurements are estimated to be 10% (2 s.d.).

### Mercury isotope nomenclature

Mercury isotope compositions are reported using nomenclature suggested by Blum and Bergquist^48^. Isotopic compositions are reported using *δ*-notation relative to the NIST SRM 3133 standard according to equation (1):


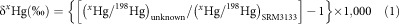


where *x* is the mass number of each Hg isotope from ^199^Hg to ^204^Hg. We use δ^202^Hg to report MDF. MIF is reported as Δ^*x*^Hg, which is defined using equation (2):


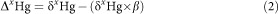


where *x* is the mass number of each Hg isotope (199, 200, 201 and 204) and *β* is the scaling constant used to estimate theoretical MDF based on kinetic mass fractionation^49^. *β* is 0.2520, 0.5024, 0.7520 and 1.493 for ^199^Hg, ^200^Hg, ^201^Hg and ^204^Hg, respectively.

### Mercury isotope measurements

Before isotope analysis, Hg was extracted and purified from samples by combustion separation using the furnace module of the Hydra II_c_ with the gold trap removed. The decomposition procedure was the same as described for the Hg concentration measurements. To trap Hg, the gas outflow containing elemental Hg was sparged directly into a freshly prepared solution of ∼10% trace metal grade H_2_SO_4_ (v/v) and ∼1% KMnO_4_ (w/w), where the Hg^0^ gas was oxidized to Hg(II). After the combustion of each sample, 50 μl of Milli-Q water was loaded into a nickel boat and combusted according to the same procedures as samples to ensure removal of any residual Hg in the furnace. During this step, the line linking the gas outflow to the sparger was also heated with a heat gun to ensure full recovery of Hg.

Aqueous solutions of NIST 3133, powders of NIST 1646a and blanks were combusted and trapped alongside samples as procedural standards and blanks. Procedural blanks were <0.02 ng g^−1^, which is <1–2% of the sample Hg. Recovery of Hg from samples and process standards was checked by neutralizing an aliquot of each solution with NH_2_OH-HCl immediately after trapping and measuring its concentration using a Tekran 2600 cold vapour atomic fluorescence spectrometer. The recoveries of samples were 99.3±10.6%, (2 s.d., *n*=35) and of procedural standards were 99.6±4.8% (2 s.d., *n*=8). The ∼10% variation in sample recoveries reflects both the uncertainty in concentration method and sample heterogeneity.

Isotopic analysis was conducted using a cold vapour multi-collector inductively coupled plasma mass spectrometer (Neptune Plus, Thermo-Finnigan) at the University of Toronto. Sample solutions were first neutralized with NH_2_OH-HCl in order to reduce KMnO_4_ and then diluted to 1–2 ng g^−1^ using a pre-neutralized 1% KMnO_4_ solution (the same matrix as samples). Hg was introduced into the plasma as Hg(0) using online SnCl_2_ reduction and Hg(0) vapour separation. To correct for instrumental mass bias, we used an internal Tl standard (NIST 997; introduced as a desolvated aerosol) and strict standard-sample bracketing with the NIST 3133 Hg standard. In addition, an in-house secondary aqueous Hg standard (J.T.Baker Chemicals) was measured at least seven times in each analytical session to determine the external reproducibility of the method. Both the NIST 3133 bracketing standards and the J.T.Baker Hg standards were prepared in the same matrix solution as samples and procedural standards. Signal concentrations and intensities of all standards and samples were matched within 10%. Isobaric interference from ^204^Pb was monitored using ^206^Pb, but was always negligible (correction never altered the calculated δ^204^Hg). On-peak blank corrections were made on all Hg and Pb masses and the Hg intensities of the blank measurements were monitored to ensure negligible carry-over and build up of Hg.

The average value of the JT Baker Hg standard over all analytical sessions was −0.60±0.09‰ for δ^202^Hg and 0.02±0.03‰ for Δ^199^Hg (2 s.d., *n*=31; Supplementary Table 2), which is consistent with previous values on this standard^50^,^51^. All samples and procedural standards were measured at least twice. Sample isotope values are reported as the mean of duplicate or triplicate measurements (Supplementary Table 1). Isotopic values obtained on the NIST 3133 procedural standards are within error of our bracketing standard with an average δ^202^Hg of 0.02±0.03‰ and Δ^199^Hg of −0.01±0.01‰ (2 s.e.m., *n*=4; Supplementary Table 2). Isotopic values for the NIST 1646a procedural standards are consistent with previously measured values for this standard in our laboratory with an average δ^202^Hg of −0.90±0.05‰ and Δ^199^Hg of 0.08±0.01‰ (2 s.e.m., *n*=4; Supplementary Table 2)^50^. We chose NIST 1646a as a procedural isotope standard for this study because it has Hg concentrations and slight MIF similar to our samples. Over the same time period in the lab, NIST 1944 was also measured by the above combustion procedure and had an average δ^202^Hg of −0.43±0.03‰ and Δ^199^Hg of 0.01±0.02‰ (2 s.e.m., *n*=5), which is within error of published values^50^. Sample errors are reported as 2 s.e.m. of sample replicates unless that value is smaller than 2 s.d. of the in-house JT Baker Hg standard. If the 2 s.e.m. of sample replicates is smaller than the 2 s.d. of the JT Baker standard, then the 2 s.d. of the JT Baker standard is used as the error for the sample.

## Additional information

**How to cite this article:** Thibodeau, A. M. *et al*. Mercury anomalies and the timing of biotic recovery following the end-Triassic mass extinction. *Nat. Commun.* 7:11147 doi: 10.1038/ncomms11147 (2016).

## Supplementary Material

Supplementary Information Supplementary Figures 1-4, Supplementary Tables 1-2 and Supplementary References.

## Figures and Tables

**Table 1:** Key geochemical parameters on all samples.

Sample ID	Depth (m)	Formation	Member	δ^13^C_org_ (‰)	TOC (%)	Hg (p.p.b.)	Hg/TOC	Δ^199^Hg (‰)	δ^202^Hg (‰)
NYC-D	66.9	Sunrise	Ferguson Hill	−24.6	0.05	8.6	188	−0.30	−0.98
NYC-C	65.4	Sunrise	Ferguson Hill	−26.5	0.04	6.2	149	NA	NA
NV ooids 2B	58.4	Sunrise	Ferguson Hill	−24.3	0.05	ND	NA	NA	NA
NVSF19	54.4	Sunrise	Ferguson Hill	−21.3	0.05	1.8	37	NA	NA
NVSF17	51.4	Sunrise	Ferguson Hill	−23.6	0.03	5.3	167	NA	NA
NVSF12A	41.4	Sunrise	Ferguson Hill	−17.2	0.04	1.5	40	NA	NA
NVSF8	35.4	Sunrise	Ferguson Hill	−28.3	0.08	14.6	195	0.01	−0.83
NVSF4	31.4	Sunrise	Ferguson Hill	−28.9	0.08	4.7	57	NA	NA
NVSF1-12	28.4	Sunrise	Ferguson Hill	−28.7	0.10	9.0	87	0.11	−1.78
NVSF1-8	24.4	Sunrise	Ferguson Hill	−27.5	0.06	7.9	130	NA	NA
NVSF1-4	20.4	Sunrise	Ferguson Hill	−29.0	0.15	5.8	40	NA	NA
TJ-1	18.5	Sunrise	Ferguson Hill	−28.3	0.16	19.4	121	−0.17	−0.90
TJ-2	18.1	Sunrise	Ferguson Hill	−27.4	0.22	23.2	105	−0.01	−1.03
TJ-3	17.7	Sunrise	Ferguson Hill	−28.3	0.14	27.9	195	0.02	−1.38
TJ-4	16.0	Sunrise	Ferguson Hill	−28.8	0.20	17.3	86	−0.03	0.12
TJ-5	14.7	Gabbs	Muller Canyon	−28.8	0.26	18.4	71	0.07	−1.25
TJ-6	14.1	Gabbs	Muller Canyon	−28.7	0.36	15.5	43	−0.02	0.23
TJ-7	13.8	Gabbs	Muller Canyon	−28.1	0.17	14.4	83	0.05	−1.00
TJ-8	12.8	Gabbs	Muller Canyon	−27.4	0.19	17.3	90	0.01	−0.66
TJ-9	12.5	Gabbs	Muller Canyon	−27.8	0.51	48.1	95	0.01	−0.44
TJ-10	11.9	Gabbs	Muller Canyon	−27.6	0.38	31.4	83	0.02	−0.79
TJ-11	10.7	Gabbs	Muller Canyon	−27.8	0.48	39.0	81	0.02	−1.26
TJ-12	10.3	Gabbs	Muller Canyon	−27.6	0.13	22.5	173	0.00	−0.56
TJ-13	9.2	Gabbs	Muller Canyon	−28.1	0.33	18.5	57	0.03	−0.41
TJ-14	8.9	Gabbs	Muller Canyon	−28.6	0.24	23.9	100	0.04	−1.17
TJ-15	8.6	Gabbs	Muller Canyon	−28.2	0.31	25.9	85	−0.05	−0.30
TJ-16	8.2	Gabbs	Muller Canyon	−28.1	0.36	46.7	128	−0.01	−0.60
TJ-17	6.8	Gabbs	Muller Canyon	−28.3	0.11	42.4	395	0.02	−0.93
TJ-18	6.6	Gabbs	Muller Canyon	−28.2	0.11	62.0	588	−0.01	−0.67
TJ-19	6.2	Gabbs	Muller Canyon	−28.1	0.26	108	410	−0.01	−0.50
TJ-20	6.2	Gabbs	Muller Canyon	−28.7	0.15	49.6	335	0.06	−0.96
TJ-21	6.0	Gabbs	Muller Canyon	−28.7	0.29	50.5	173	−0.01	−0.16
TJ-22	6.2	Gabbs	Muller Canyon	−29.0	0.17	44.6	266	−0.01	−0.70
TJ-23	5.8	Gabbs	Muller Canyon	−29.4	0.36	64.1	177	0.03	−0.67
TJ-24	5.6	Gabbs	Muller Canyon	−29.4	0.32	52.1	164	0.02	−0.21
TJ-25	5.6	Gabbs	Muller Canyon	−29.4	0.28	46.7	167	0.02	−0.47
TJ-26	5.1	Gabbs	Muller Canyon	−29.4	0.20	25.5	129	0.03	−0.55
TJ-27	4.6	Gabbs	Muller Canyon	−29.8	0.14	24.5	173	0.04	−0.97
TJ-28	4.2	Gabbs	Muller Canyon	−29.1	0.13	24.7	185	0.00	−0.16
TJ-29	3.4	Gabbs	Muller Canyon	−29.7	0.39	47.1	120	−0.02	−0.62
TJ-30	2.6	Gabbs	Muller Canyon	−29.5	0.36	38.3	106	−0.01	−0.66
TJ-31	2.3	Gabbs	Muller Canyon	−28.6	0.36	16.5	45	−0.01	0.05
TJ-32	−0.2	Gabbs	Mt Hyatt	−28.8	0.18	6.7	37	NA	NA
TJ-33	−0.9	Gabbs	Mt Hyatt	−28.6	0.29	14.0	48	0.08	0.07
TJ-34	−1.1	Gabbs	Mt Hyatt	−28.4	0.28	9.2	32	0.01	−0.20
TJ-35	−2.0	Gabbs	Mt Hyatt	−28.5	0.14	9.5	67	−0.08	−0.56

NA, not analysed; ND, not detected; TOC, total organic carbon.

δ^13^C_org_ values are relative to the Vienna Pee Dee Belemnite. Depths are measured in metres (m) relative to the Mt Hyatt/Muller Canyon contact.
