# Supplementary material for: Mercury anomalies and the timing of biotic recovery following the end-Triassic mass extinction
Source: Nat Commun. 2016 Apr 6;7:11147. doi: 10.1038/ncomms11147 (PMC4823824; doi:10.1038/ncomms11147)
Supplement: Supplementary Information — Supplementary Figures 1-4, Supplementary Tables 1-2 and Supplementary References. [file ncomms11147-s1.pdf]

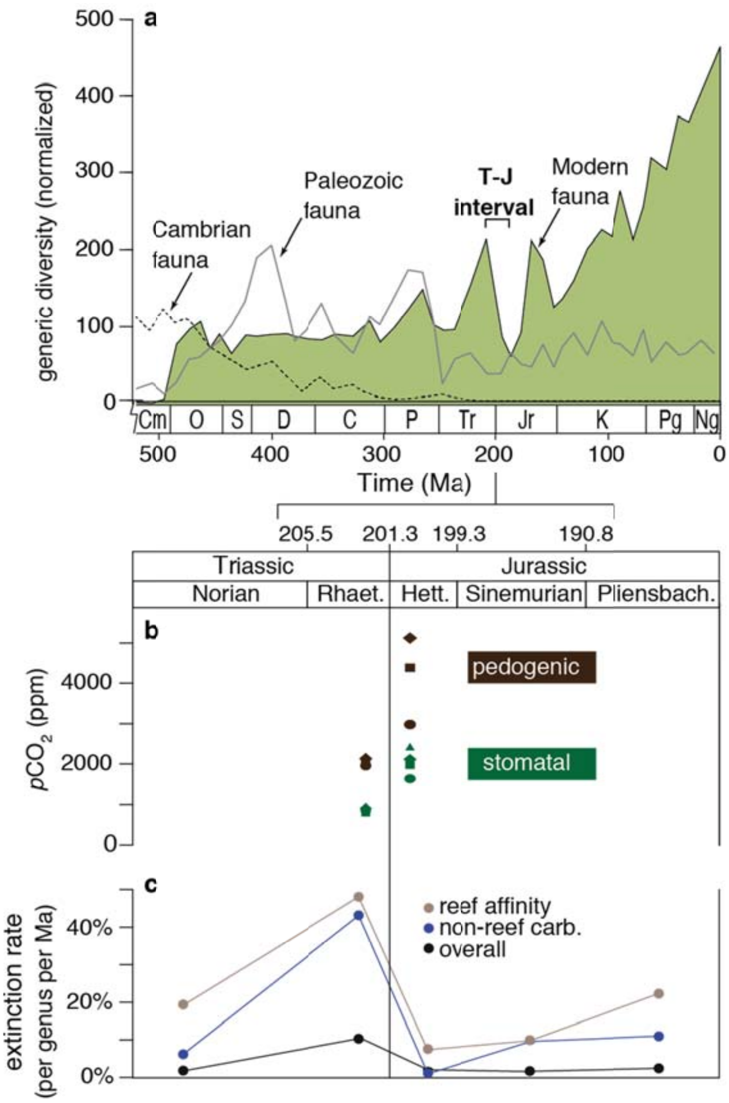

Supplementary Figure 1: Generic diversity, *p*CO_2, and extinction rates across the T-J interval. a) Generic diversity of the Cambrian, Paleozoic, and Modern Faunas, highlighting that the T-J extinction was particularly devastating to the Modern Fauna.[1] b) Summary of *p*CO_2 levels across the T-J from stomatal proxies (green) and pedogenic proxies (brown), after Martindale *et al.*[2] and references therein. c) Extinction rate across the T-J interval, highlighting preferential extinction of fauna associated with carbonate environments and reefs, after Kiessling *et al.*[3]

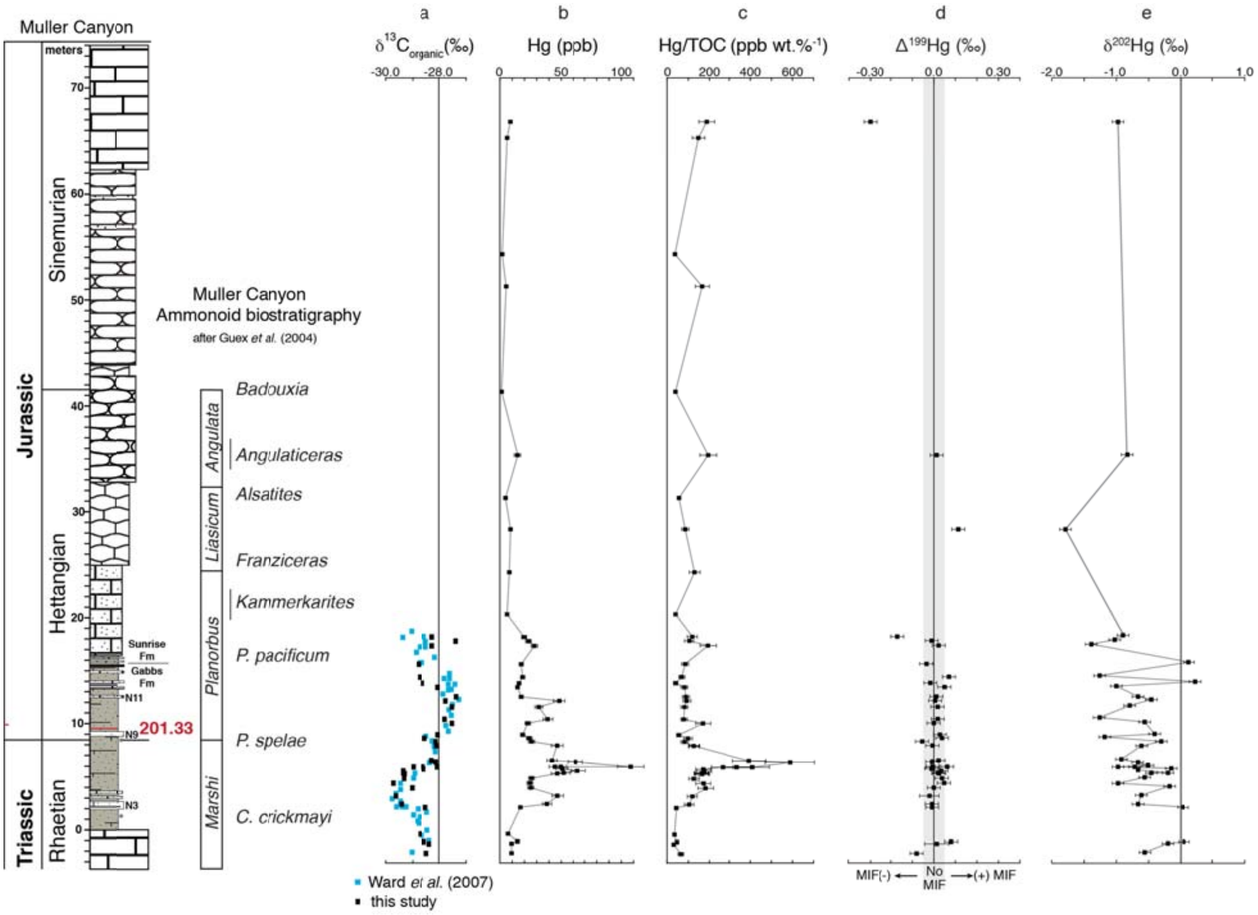

Supplementary Figure 2: **Extended lithology, ammoniod biostratigraphy, and geochemical data for the Muller Canyon succession**. Panels show (a) $\delta^{13}C_{org}$, (b) Hg, (c) Hg/TOC, (d) Δ^{199}Hg, and (e) δ^{202}Hg for Muller Canyon, Nevada, in association with ammonoid biostratigraphy and lithology. Marker beds and ammonoid biostratigraphy are after Guex *et al*.[4] and ash date is from Schoene *et al*.[5] Key to lithology is same as shown in Fig. 3. Panel (b) compares $\delta^{13}C_{org}$ measurements from this study (black dots) with data from Ward *et al*.[6] corrected for the duplication by the fault (blue dots, see Supplementary Figure 4). Vertical grey bar in panel (d) as well as error bars on Hg, Hg/TOC, and Δ^{199}Hg measurements are as described in Fig. 2. Error bars on δ^{202}Hg are as reported in Supplementary Table 2 and represent either 2 s.e.m. of sample replicates or 2 s.d. of the JT Baker standard, whichever is higher (see Methods for further explanation).

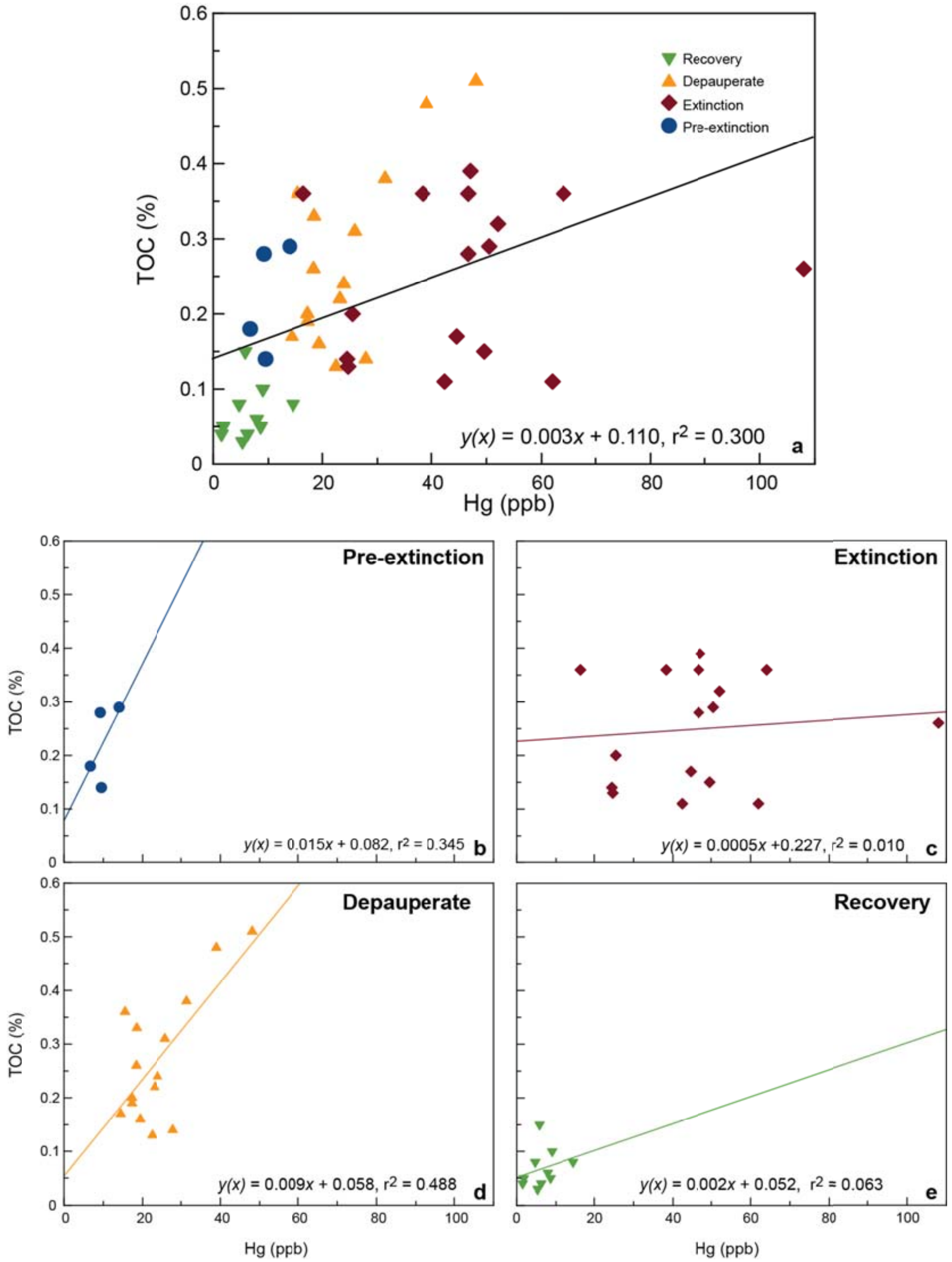

Supplementary Figure 3: Cross plots of percent total organic carbon (% TOC) vs. Hg concentration (ppb) for the Muller Canyon succession by key intervals. a) all data; b) data from the Mt Hyatt member of the Gabbs Formation (pre-extinction); c) data from the extinction interval; d) data from the depauperate interval; e) data from the first recovery interval.

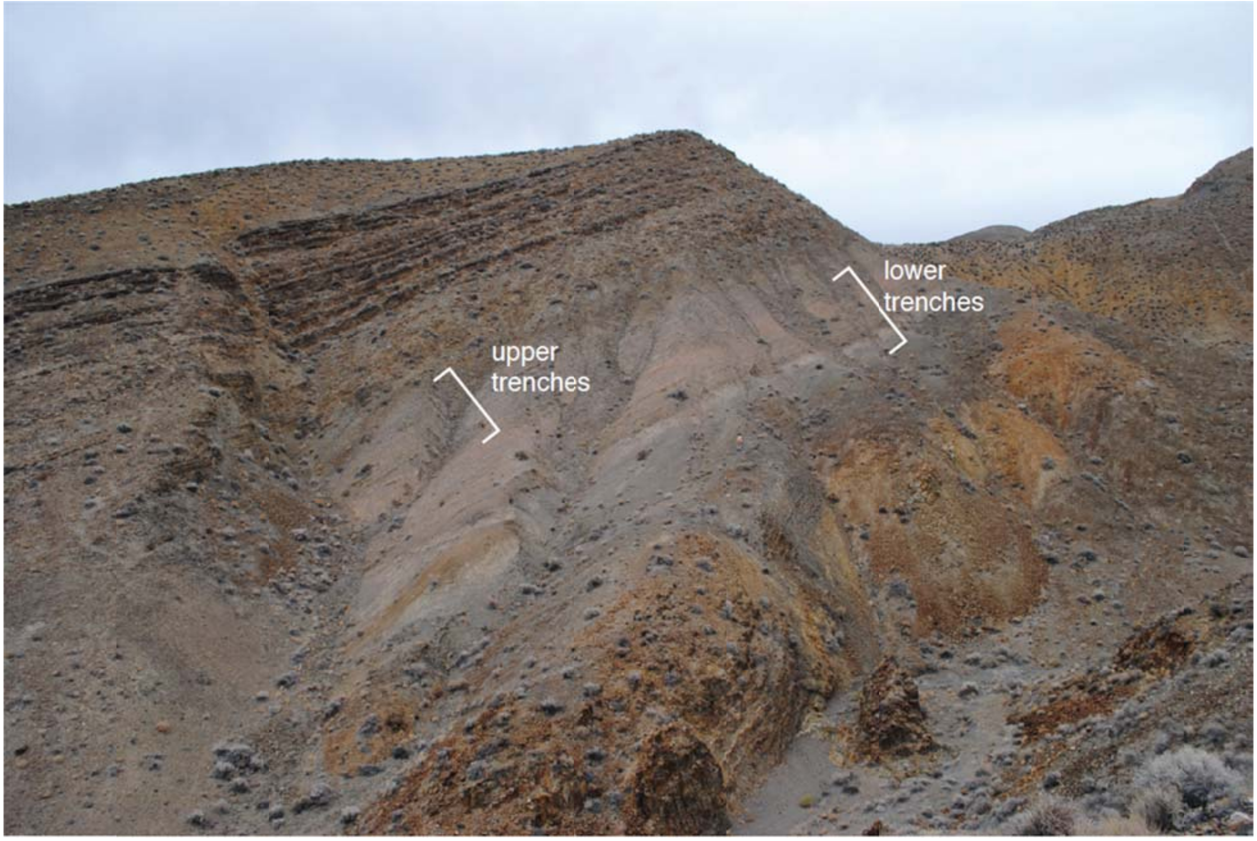

Supplementary Figure 4: Sampling sites on Ferguson Hill, Muller Canyon, Gabbs Valley Range, Nevada. Samples were collected at the meter to sub-meter scale from trenches excavated in order to obtain the most un-weathered specimens. The fault that duplicates the white layer in the lower part of the succession, visible downslope from the upper trenches, was avoided.

Supplementary Table 1: Mercury concentrations and isotopic compositions of all rock samples

Sample ID	Hg (ppb)	n	$\delta^{204}Hg$	2σ	$\delta^{202}Hg$	2σ	$\delta^{201}Hg$	2σ	$\delta^{200}Hg$	2σ	$\delta^{199}Hg$	2σ	$\Delta^{204}Hg$	2σ	$\Delta^{201}Hg$	2σ	$\Delta^{200}Hg$	2σ	$\Delta^{199}Hg$	2σ
**NYC-D**	8.6	2	-1.37	0.13	-0.98	0.09	-0.96	0.09	-0.55	0.05	-0.54	0.04	0.09	0.04	-0.23	0.04	-0.05	0.02	-0.30	0.03
**NSF8**	14.7	2	-1.23	0.13	-0.83	0.09	-0.65	0.09	-0.41	0.05	-0.20	0.04	0.00	0.03	-0.03	0.04	0.01	0.02	0.01	0.03
**NSF1-12**	9.00	2	-2.68	0.14	-1.78	0.09	-1.30	0.09	-0.85	0.05	-0.34	0.04	-0.03	0.06	0.04	0.04	0.04	0.02	0.11	0.03
**TJ-1**	19.4	2	-1.31	0.13	-0.90	0.09	-0.83	0.09	-0.47	0.05	-0.40	0.04	0.04	0.03	-0.15	0.04	-0.02	0.02	-0.17	0.03
**TJ-2**	23.2	2	-1.54	0.13	-1.03	0.09	-0.80	0.09	-0.52	0.05	-0.27	0.04	0.00	0.03	-0.02	0.04	-0.01	0.02	-0.01	0.03
**TJ-3**	27.9	3	-2.08	0.13	-1.38	0.09	-1.05	0.09	-0.68	0.05	-0.33	0.04	-0.02	0.03	-0.02	0.04	0.01	0.02	0.02	0.03
**TJ-4**	17.3	2	0.18	0.13	0.12	0.09	0.06	0.09	0.06	0.05	0.00	0.04	-0.01	0.05	-0.04	0.04	0.00	0.02	-0.03	0.03
**TJ-5**	18.4	2	-1.89	0.13	-1.25	0.09	-0.93	0.09	-0.62	0.05	-0.24	0.04	-0.03	0.04	0.01	0.04	0.01	0.02	0.07	0.03
**TJ-6**	15.5	2	0.33	0.13	0.23	0.09	0.13	0.09	0.12	0.05	0.04	0.04	-0.02	0.04	-0.04	0.04	0.01	0.03	-0.02	0.03
**TJ-7**	14.4	2	-1.53	0.13	-1.00	0.09	-0.75	0.09	-0.47	0.05	-0.20	0.04	-0.04	0.04	0.00	0.04	0.03	0.02	0.05	0.03
**TJ-8**	17.3	2	-0.99	0.13	-0.66	0.09	-0.52	0.09	-0.31	0.05	-0.15	0.04	-0.01	0.07	-0.02	0.04	0.02	0.02	0.01	0.03
**TJ-9**	48.1	2	-0.69	0.13	-0.44	0.09	-0.36	0.09	-0.22	0.05	-0.11	0.04	-0.03	0.03	-0.02	0.04	0.01	0.02	0.01	0.03
**TJ-10**	31.4	2	-1.19	0.13	-0.79	0.09	-0.61	0.09	-0.38	0.05	-0.18	0.04	-0.01	0.04	-0.01	0.04	0.02	0.02	0.02	0.03
**TJ-11**	39.0	2	-1.89	0.13	-1.26	0.09	-0.95	0.09	-0.60	0.05	-0.30	0.04	0.00	0.07	0.00	0.04	0.03	0.02	0.02	0.03
**TJ-12**	22.5	2	-0.83	0.13	-0.56	0.09	-0.45	0.09	-0.26	0.05	-0.14	0.04	0.01	0.03	-0.03	0.04	0.02	0.02	0.00	0.03
**TJ-13**	18.5	2	-0.62	0.13	-0.41	0.09	-0.30	0.09	-0.19	0.05	-0.08	0.04	-0.01	0.05	0.01	0.04	0.02	0.02	0.03	0.03
**TJ-14**	23.9	2	-1.76	0.13	-1.17	0.09	-0.88	0.09	-0.58	0.05	-0.26	0.04	-0.01	0.03	0.00	0.04	0.01	0.02	0.04	0.03
**TJ-15**	25.9	2	-0.46	0.13	-0.30	0.09	-0.28	0.09	-0.15	0.05	-0.13	0.04	-0.01	0.03	-0.06	0.04	0.00	0.02	-0.05	0.03
**TJ-16**	46.7	2	-0.89	0.13	-0.60	0.09	-0.47	0.09	-0.29	0.05	-0.16	0.04	0.01	0.03	-0.02	0.04	0.01	0.02	-0.01	0.03
**TJ-17**	42.4	2	-1.40	0.14	-0.93	0.09	-0.69	0.09	-0.45	0.08	-0.21	0.05	-0.02	0.03	0.01	0.04	0.02	0.04	0.02	0.03
**TJ-18**	62.0	2	-1.02	0.13	-0.67	0.09	-0.53	0.09	-0.33	0.05	-0.18	0.04	-0.01	0.03	-0.02	0.04	0.01	0.02	-0.01	0.03
**TJ-19**	108	2	-0.78	0.13	-0.51	0.09	-0.42	0.09	-0.24	0.05	-0.14	0.04	-0.03	0.03	-0.04	0.04	0.01	0.02	-0.01	0.03
**TJ-19-R**		3	-0.76	0.13	-0.49	0.09	-0.38	0.09	-0.23	0.05	-0.12	0.04	-0.03	0.03	-0.02	0.04	0.01	0.02	0.00	0.03
**TJ-20**	49.6	2	-1.46	0.13	-0.96	0.09	-0.71	0.09	-0.47	0.05	-0.18	0.04	-0.03	0.03	0.02	0.04	0.01	0.03	0.06	0.03
**TJ-21**	50.5	2	-0.28	0.13	-0.16	0.09	-0.15	0.09	-0.06	0.05	-0.05	0.04	-0.05	0.05	-0.03	0.04	0.02	0.02	-0.01	0.03
**TJ-22**	44.6	2	-1.06	0.13	-0.70	0.09	-0.55	0.09	-0.36	0.05	-0.18	0.04	-0.01	0.03	-0.02	0.04	0.00	0.02	-0.01	0.03

Supplementary Table 1 *(continued)*

Sample ID	Hg (ppb)	n	δ^{204}Hg	2σ	δ^{202}Hg	2σ	δ^{201}Hg	2σ	δ^{200}Hg	2σ	δ^{199}Hg	2σ	Δ^{204}Hg	2σ	Δ^{201}Hg	2σ	Δ^{200}Hg	2σ	Δ^{199}Hg	2σ
**TJ-23**	64.1	2	-1.01	0.13	-0.67	0.09	-0.51	0.09	-0.32	0.05	-0.14	0.04	-0.01	0.08	0.00	0.04	0.01	0.03	0.03	0.03
**TJ-24**	52.1	2	-0.32	0.15	-0.21	0.09	-0.17	0.09	-0.07	0.05	-0.03	0.04	-0.01	0.03	-0.01	0.06	0.03	0.02	0.02	0.03
**TJ-25**	46.7	2	-0.71	0.13	-0.47	0.09	-0.37	0.09	-0.22	0.05	-0.09	0.04	-0.01	0.04	-0.02	0.04	0.02	0.02	0.02	0.03
**TJ-26**	25.5	2	-0.81	0.13	-0.55	0.09	-0.39	0.09	-0.27	0.05	-0.10	0.04	0.00	0.03	0.02	0.04	0.00	0.02	0.03	0.03
**TJ-27**	24.5	2	-1.48	0.13	-0.97	0.09	-0.72	0.09	-0.45	0.05	-0.20	0.04	-0.04	0.03	0.01	0.04	0.03	0.02	0.04	0.03
**TJ-28**	24.7	2	-0.26	0.13	-0.16	0.09	-0.14	0.09	-0.07	0.05	-0.04	0.04	-0.01	0.03	-0.01	0.04	0.02	0.02	0.00	0.03
**TJ-29**	47.1	2	-0.98	0.13	-0.62	0.09	-0.49	0.09	-0.30	0.05	-0.18	0.05	-0.05	0.03	-0.03	0.04	0.01	0.02	-0.02	0.04
**TJ-30**	38.3	2	-1.03	0.13	-0.66	0.09	-0.53	0.09	-0.33	0.05	-0.18	0.04	-0.04	0.03	-0.04	0.04	0.00	0.03	-0.01	0.03
**TJ-31**	16.5	2	0.08	0.13	0.05	0.09	0.03	0.09	0.04	0.06	0.01	0.04	0.00	0.03	-0.01	0.04	0.02	0.03	-0.01	0.03
**TJ-33**	14.0	2	0.08	0.13	0.07	0.09	0.08	0.09	0.04	0.05	0.09	0.04	-0.02	0.03	0.03	0.04	0.00	0.02	0.08	0.03
**TJ-34**	9.2	2	-0.34	0.13	-0.20	0.09	-0.13	0.09	-0.08	0.05	-0.04	0.04	-0.04	0.03	0.03	0.04	0.02	0.04	0.01	0.05
**TJ-35**	9.5	2	-0.81	0.13	-0.56	0.09	-0.48	0.09	-0.29	0.05	-0.22	0.04	0.02	0.03	-0.06	0.04	-0.01	0.04	-0.08	0.03

Note: Sample concentrations have an error of 10% (2 s.d.) based on the reproducibility of external standards and several samples (See Methods). Each sample solution was measured at least twice for Hg isotopic ratios. Values given here are averages of all analytical replicates. Errors on the isotopic measurements are either 2 s.e.m. of analytical replicates or 2 s.d. of the in-house JT Baker Hg standard (see Supplementary Table 2), whichever is higher.

Supplementary Table 2: Hg isotopic compositions of procedural and analytical standards

Sample ID	$\delta^{204}Hg$	2SE	$\delta^{202}Hg$	2SE	$\delta^{201}Hg$	2SE	$\delta^{200}Hg$	2SE	$\delta^{199}Hg$	2SE	$\Delta^{204}Hg$	2SE	$\Delta^{201}Hg$	2SE	$\Delta^{200}Hg$	2SE	$\Delta^{199}Hg$	2SE
NIST 3133 Session1	0.01	0.07	-0.01	0.06	0.00	0.04	-0.02	0.06	-0.01	0.02	0.03	0.02	0.01	0.00	-0.01	0.03	-0.01	0.00
NIST 3133 Session2	0.05	0.00	0.04	0.00	0.04	0.02	0.02	0.03	0.01	0.01	-0.01	0.00	0.01	0.02	0.00	0.03	0.00	0.01
NIST 3133 Session3	0.03	0.06	0.02	0.04	0.00	0.02	0.01	0.03	0.01	0.04	0.00	0.00	-0.02	0.01	0.00	0.01	0.00	0.03
NIST 3133 Session4	-0.04	0.10	-0.01	0.02	0.01	0.02	-0.01	0.01	-0.02	0.00	-0.02	0.07	0.01	0.00	0.00	0.00	-0.02	0.01
Mean NIST 3133 (all sessions)	0.01	0.04	0.01	0.02	0.01	0.02	0.00	0.02	0.00	0.02	0.00	0.02	0.00	0.01	0.00	0.01	-0.01	0.01
NIST 1646a Session 1	-1.39	0.11	-0.90	0.08	-0.65	0.01	-0.43	0.03	-0.15	0.01	-0.05	0.01	0.03	0.05	0.02	0.01	0.08	0.01
NIST 1646a Session 2	-1.33	0.07	-0.86	0.05	-0.61	0.05	-0.40	0.04	-0.12	0.01	-0.05	0.00	0.04	0.02	0.03	0.02	0.09	0.00
NIST 1646a Session 3	-1.48	0.04	-0.98	0.02	-0.70	0.08	-0.46	0.04	-0.17	0.01	-0.02	0.01	0.04	0.06	0.03	0.03	0.08	0.02
NIST 1646a Session 4	-1.37	0.08	-0.88	0.05	-0.63	0.06	-0.41	0.04	-0.14	0.04	-0.06	0.00	0.02	0.02	0.03	0.02	0.08	0.03
Mean NIST 1646a (all sessions)	-1.39	0.06	-0.90	0.05	-0.65	0.04	-0.43	0.03	-0.14	0.02	-0.04	0.01	0.03	0.01	0.03	0.01	0.08	0.01
**Sample ID**	**$\delta^{204}Hg$**	**2SD**	**$\delta^{202}Hg$**	**2SD**	**$\delta^{201}Hg$**	**2SD**	**$\delta^{200}Hg$**	**2SD**	**$\delta^{199}Hg$**	**2SD**	**$\Delta^{204}Hg$**	**2SD**	**$\Delta^{201}Hg$**	**2SD**	**$\Delta^{200}Hg$**	**2SD**	**$\Delta^{199}Hg$**	**2SD**
JTBaker Hg Std (all sessions, n=31)	-0.91	0.13	-0.60	0.09	-0.46	0.09	-0.29	0.05	-0.13	0.04	-0.01	0.03	0.00	0.04	0.01	0.02	0.02	0.03

Note: NIST 3133 and NIST 1646a are procedural standards processed with each batch of samples (See Methods). The JT Baker Standard is an in-house secondary standard that was measured seven or more times in each analytical session.

Supplementary References

1. Alroy, J. The shifting balance of diversity among major marine animal groups. *Science* **329,** 1191–1194 (2010).
2. Martindale, R. C., Berelson, W. M., Corsetti, F. A., Bottjer, D. J. & West, A. J. Constraining carbonate chemistry at a potential ocean acidification event (the Triassic-Jurassic boundary) using the presence of corals and coral reefs in the fossil record. *Palaeogeography, Palaeoclimatology, Palaeoecology* **350-352,** 114–123 (2012).
3. Kiessling, W., Aberhan, M., Brenneis, B. & Wagner, P. J. Extinction trajectories of benthic organisms across the Triassic–Jurassic boundary. *Palaeogeography, Palaeoclimatology, Palaeoecology* **244,** 201–222 (2007).
4. Guex, J., Bartolini, A., Atudorei, V. & Taylor, D. High-resolution ammonite and carbon isotope stratigraphy across the Triassic–Jurassic boundary at New York Canyon (Nevada). *Earth and Planetary Science Letters* **225,** 29–41 (2004).
5. Schoene, B., Guex, J., Bartolini, A., Schaltegger, U. & Blackburn, T. J. Correlating the end-Triassic mass extinction and flood basalt volcanism at the 100 ka level. *Geology* **38,** 387–390 (2010).
6. Ward, P. D. *et al.* The organic carbon isotopic and paleontological record across the Triassic–Jurassic boundary at the candidate GSSP section at Ferguson Hill, Muller Canyon, Nevada, USA. *Palaeogeography, Palaeoclimatology, Palaeoecology* **244,** 281–289 (2007).
